# Prevalence and Diversity of Avian Haematozoan Parasites in Wetlands of Bangladesh

**DOI:** 10.1155/2014/493754

**Published:** 2014-01-21

**Authors:** Rubayet Elahi, Ausraful Islam, Mohammad Sharif Hossain, Khaja Mohiuddin, Andrea Mikolon, Suman Kumer Paul, Parviez Rana Hosseini, Peter Daszak, Mohammad Shafiul Alam

**Affiliations:** ^1^International Centre for Diarrhoeal Disease Research Bangladesh (icddr,b), Dhaka 1212, Bangladesh; ^2^Department of Neurobiology and Developmental Sciences, University of Arkansas for Medical Sciences (UAMS), Little Rock, AR 72205, USA; ^3^EcoHealth Alliance, New York, NY 10001, USA

## Abstract

The parasites of genera *Haemoproteus, Plasmodium, *and *Leucocytozoon* are well-known avian haematozoa and can cause declined productivity and high mortality in wild birds. The objective of the study was to record the prevalence of haematozoan parasites in a wide range of wetland birds in Bangladesh. Six species of *Haemoproteus*, seven species of *Plasmodium*, one unidentified species of *Leucocytozoon*, and one unidentified microfilaria of the genus *Paronchocerca* were found. Data on the morphology, size, hosts, prevalence, and infection intensity of the parasites are provided. The overall prevalence among the birds was 29.5% (95 out of 322 birds). Of those, 13.2% (42 of 319) of birds were infected with *Haemoproteus* spp., 15.1% with *Plasmodium* spp. (48 of 319) and 0.6% with *Leucocytozoon* spp. (2 of 319). Two birds were positive for both *Haemoproteus* sp. and *Plasmodium* sp. A single resident bird, *Ardeola grayii*, was found positive for an unidentified microfilaria. Prevalence of infection varied significantly among different bird families. Wild birds of Bangladesh carry several types of haematozoan parasites. Further investigation with a larger sample size is necessary to estimate more accurately the prevalence of haematozoan parasites among wild birds as well as domestic ducks for better understanding of the disease ecology.

## 1. Introduction

*Haemoproteus, Plasmodium, *and *Leucocytozoon *are well-known genera of avian haematozoa. [1–3]. Biting midges, louse flies, black flies, and mosquitoes are the vectors that transmit these parasites [3–5]. Though the importance of bird haematozoans remains undervalued, declined productivity of birds and high mortality due to these parasitic infections have been reported [3]. Most infections with parasites of genus *Haemoproteus* produce subclinical infections. Liver, spleen, kidneys, and gizzards become enlarged [6–8]. *Haemoproteus* can also parasitize in the lungs [9]. In some birds, anemia, anorexia, and depression have also been reported [3]. Parasites of the *Plasmodium *genus cause avian malaria which has sublethal effects on host fitness. The most significant impact is long-term effect on the reproductive system of the host causing population decrease [10]. *Leucocytozoon* typically causes anemia and enlargement of liver and spleen [3, 7]. Earlier research from South and Southeast Asia has reported the prevalence and geographical distribution of avian haematozoa [11–15]. In 2005, five species of *Plasmodium *genus, one of *Haemoproteus *genus, and two unidentified microfilariae of different birds were reported from Pakistan [16]. Distributions of *Haemoproteus *spp. and *Plasmodium *spp. in rock pigeons (*Columba livia*) from different areas of India have been described with their prevalence and seasonal variations [17].

Of 690 known bird species of Bangladesh, 380 are resident and 310 are migratory (209 winter visitors, 11 summer visitors, and 90 species vagrants) [18]. To date no information is available on haematozoan parasites in wild birds of Bangladesh although *Leucocytozoon *spp. from domestic ducks [6] and *Haemoproteus *spp. in domestic pigeons [7] have been reported recently. As part of a survey for influenza virus in wild birds, this study was conducted to identify the haematozoan parasites of various wild birds from different areas of Bangladesh. The objective of the study was to record the prevalence of haematozoan parasites in a wide range of wetland birds. Morphology of parasites and intensity of invasion were also recorded.

## 2. Materials and Methods

**Figure 1:**
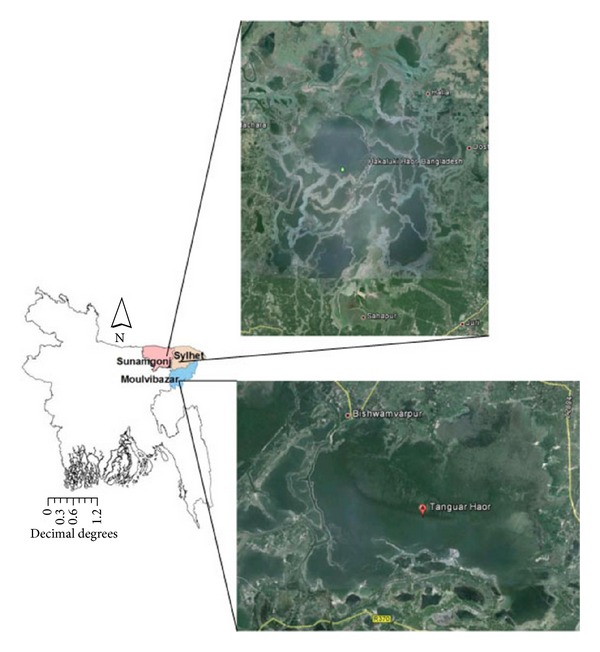
Study sites.

A total of 322 wild birds were studied belonging to 48 species (15 families and four orders) from January 2011 to March 2011. The birds were sampled from two wetland sites in Bangladesh: Hakaluki haor (N 21°33′′698, E 091°51′′682) in Sylhet and Moulvibazar districts (212 birds) and Tanguar haor (N 25°08.794′, E 091°04.088′) in Sunamganj district (110 birds) (Figure 1). Hakaluki haor and Tanguar haor are seasonal water bodies located in northern Bangladesh which dry up during winter when they provide habitat for resident and migratory wild birds. Mist nets and noose traps were used for bird capicture. Blood was drawn from jugular or tarsal vein of the bird. Species identification and age determination were made using the description given by Grimmett et al. [19]. All of the birds were marked using metal rings with unique identification numbers at the tarsus and released after sampling at the site of capture.

From each bird, typically three (but sometimes two) thin blood smears were prepared on clean, grease-free slides. All slides were fixed in absolute methanol for one minute in the field. The fixed smears were then stained with 20% Giemsa stain and were observed under 400x and 1000x magnification by skilled parasitologists. Identification of the haematozoans was performed using the taxonomic description of Valkiunas [3] and Bartlett [4]. If any parasite was found within 100 fields of microscopic observation, the slide was considered as positive; otherwise it was considered as negative. All parasites in 100 microscope fields at 1000x magnification were counted to calculate the intensity of invasion of the parasites. The nuclear displacement ratio (NDR), an index of lateral displacement of the erythrocyte nucleus by the parasite [20], was measured following the description of Valkiunas [3]. Slides of each parasite identified up to species level have been deposited in the US National Parasite Collection (USNPC), Beltsville, MD, USA (accession numbers are in the supplementary file 1; see Supplementary Material available online at http://dx.doi.org/10.1155/2014/493754).

The birds were divided into 2 groups: migratory and resident birds. All measurements in the text and the tables are presented in micrometers and given as mean ± standard deviation. Intensity of invasion was calculated per 100 microscopic fields. Statistical Package for the Social Sciences (SPSS) v 17 (SPSS Inc., USA) was used to analyze the data. A two-tailed Fisher's exact test was used to find the correlation of prevalence within bird families and parasite genus. To indicate statistical significance, a *P* value less than 0.05 was used. 

## 3. Results

### 3.1. Prevalence and Intensity of Infection

The overall prevalence of infection of the birds studied was 29.5% (95/322 birds). Among those, 13.2% (42/319) prevalence of infection was recorded for *Haemoproteus* spp., 15.1% for *Plasmodium *spp. (48/319), and 0.6% for *Leucocytozoon* spp. (2/319). Two birds were positive for parasites of both *Haemoproteus *and *Plasmodium *genera. A single slide was positive for unidentified microfilariae of genus *Paronchocerca* from *Ardeola grayii*, a resident bird (Table 1).

The prevalence of infection for the three genera varied considerably amid different families of birds (Table 1). Among the families with sufficient sample size (*n* > 20), the highest prevalence was found in the family Laridae: 44.1% (15 of 44 birds) for genus *Haemoproteus*; in the family Scolopacidae: 20.8% (5 of 24) for *Plasmodium* genus; in the family Dendrocygnidae: 2.3% (1 of 43) for *Leucocytozoon *genus. 

The intensity of invasion varied across different parasite genera. For parasites of *Haemoproteus *genus it ranged from 2 to 43, mostly being below 20 parasites per 100 microscopic fields. The lowest intensity of invasion for parasites of *Plasmodium* genus was 2, while the highest was 18 parasites per 100 microscopic fields, mostly being less than 10. Parasites of *Leucocytozoon *genus had 4-5 parasites per 100 microscopic fields, while the only bird infected with microfilariae had only 3 parasites per 100 microscopic fields.

In the present study 72.4% (*n* = 233) of the birds studied were migrant and 27.6% (*n* = 89) were resident; among these 33.5% (*n* = 78) of migrant birds and 19.1% (*n* = 17) of resident birds were positive for any of the parasites of genus *Haemoproteus, Plasmodium, or Leucocytozoon*. These parasites were more commonly found in migratory birds (*P* < 0.05). 15.9% (*n* = 37) of migrant birds and 5.7% (*n* = 5) of resident birds were positive for *Haemoproteus* genus, while 16.8% (*n* = 39) of migrant birds were positive for *Plasmodium *genus in contrast to 10.3% (*n* = 9) of resident birds. Migratory birds were significantly preferred by parasites of *Haemoproteus *(*P* < 0.05), but for parasites of *Plasmodium, *there was no significant preference. One migrant bird and one resident bird were found positive for parasites of genus *Leucocytozoon *spp. One of each migrant and resident bird had mixed infection by parasites of genera *Haemoproteus *and *Plasmodium*. There was no significant difference in the infection rates among the study sites.

### 3.2. Description of Species

Six species of *Haemoproteus *and seven species of *Plasmodium *were identified. *Haemoproteus *parasites from four birds belonging to two different species were identified up to subgenus level, subgenus *Parahaemoproteus*. *Plasmodium *parasites from five birds of three species were identified only up to subgenus level, subgenus *Haemoamoeba *and subgenus *Giovannolaia*. All the *Leucocytozoon* (*n* = 2) parasites were identified up to genus level. A single bird was found to be infected with unidentified microfilarae species of genus *Paronchocerca*. 

#### 3.2.1. Genus *Haemoproteus*, Kruse, 1890

*(1) Haemoproteus (Parahaemoproteus) pastoris*, Mello, 1935

*Morphology.* A single bird was infected with the macrogametocyte of *H. pastoris*. The outline of the gametocytes was amoeboid, adhering to the erythrocyte nucleus and envelope filling the erythrocyte up to their poles. They displaced the erythrocyte nucleus slightly. The measurement of the macrogametocytes was 14.575 ± 0.4 *μ*m ×  4.12 ± 0.3 *μ*m. The nucleus of the macrogametocyte was compact and in subcentral position measuring 3.15 ± 0.02 *μ*m ×  2.575 ± 0.1 *μ*m. 9 to 17 small roundish pigment granules were seen randomly scattered in the cytoplasm. The NDR was 0.5 ± 0.2. Invasion intensity was found to be 17 parasites per 100 microscopic fields.

*Host*. *Sturnus contra* (Asian Pied Starling). 

*Locality*. Hakaluki haor (*n* = 1).

*(2) Haemoproteus (Parahaemoproteus) scolopaci, *Galli-Valerio, 1929

*Morphology*. A single bird was found to be infected with the macrogametocyte of *H. scolopaci*. The gametocyte was oval with amoeboid outline and was slightly appressed with the host erythrocyte envelope and nucleus, completely encircling the erythrocyte nucleus and occupying all available cytoplasmic space in the erythrocytes. The nucleus was slightly displaced laterally and the host cell was hypertrophied. The NDR was 0.66 ± 0.03. The measurement of the gametocyte was 14.575 ± 1.47 *μ*M ×  3.575 ± 0.515 *μ*M. Parasite nucleus was centrally situated with the measurement of 3.09 ± 0.3 *μ*M ×  2.57 ± 0.03 *μ*M. The cytoplasm of the gametocyte was homogenous in appearance and contained small roundish pigment granules. The intensity of invasion was found to be 13 parasites per 100 microscopic fields.

*Host*. *Charadrius dubius *(Little Ringed Plover).

*Locality*. Hakaluki haor (*n* = 1).

*(3) Haemoproteus (Parahaemoproteus) plataleae, *Mello, 1935

*Morphology*. Both macro- and microgametocytes of *H. plataleae *were found in 17 birds of two species. Two birds were infected with both gametes. Both the gametocytes markedly encircled the erythrocyte nucleus and appressed closely with the host cell envelope.

**Figure 2:**
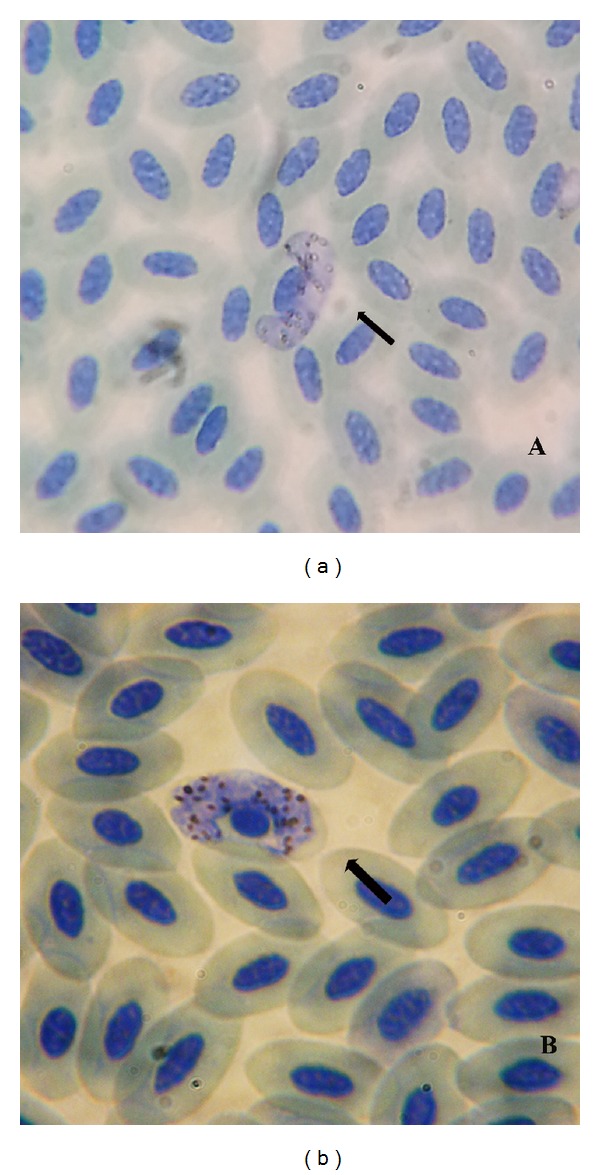
(a) Macrogametocyte of *H. plataleae.* (b) Macrogametocyte of *H. nettionis. *

The macrogametocytes were dumbbell-shaped with a measurement of 15.5 ± 1.65 *μ*M × 4.83 ± 0.88 *μ*M (Figure 2(a)). The nucleus of the parasite was in median position with the measurement of 4.37 ± 1.04 *μ*M ×  3.46 ± 0.95 *μ*M, frequently laid free in the cytoplasm. The cytoplasm was coarsely granular with 17 to 49 (34 ± 9.06) small roundish pigment granules scattered randomly in the cytoplasm. Infected erythrocytes were slightly hypertrophied. Invasion intensity per 100 microscopic fields was 4 to 28. The NDR was 0.4 ± 0.25.

The microgametocytes were dumbbell-shaped with amoeboid outline and measured 12.45 ± 2.50 *μ*M ×  4.50 ± 0.78 *μ*M. The nucleus of the parasite was in submedian position and was also laid free in the cytoplasm like macrogametocyte and measured 3.60 ± 0.73 *μ*M ×  2.83 ± 0.7 *μ*M. The cytoplasm contained more pigment granules than macrogametocytes. 10 to 41 microgametocytes were seen per 100 microscopic fields. The NDR was 0.4 ± 0.01.

*Hosts*. *Larus ridibundus *(Black headed gull) and *Ardeola grayii* (Indian pond heron).

*Locality*. Hakaluki haor (*n* = 12), Tanguar haor (*n* = 5).

*(4) Haemoproteus (Parahaemoproteus) greineri*, Bennett, Turner and Whiteway, 1984

*Morphology*. A total of 15 birds of seven species were found to be infected with *H. greineri* by both macro- and microgametocytes. Both gametocytes were dumbbell-shaped. The nucleus of the host erythrocytes was enclosed slightly by the gametocytes. Some host cells were hypertrophied.

The macrogametocytes were closely appressed with both host erythrocyte nucleus and envelope. The macrogametocytes measured 15.10 ± 1.02 *μ*M ×  4.47 ± 0.63 *μ*M. The nucleus was compact, comparatively small, and submedian in position measuring 4.74 ± 1.54 *μ*M ×  3.14 ± 0.92 *μ*M. 18–32 (24 ± 5.4) small roundish pigment granules were randomly scattered throughout the parasite cytoplasm. The NDR was 0.66 ± 0.14. The invasion intensity ranged from 11 to 39 parasites per 100 microscopic fields.

The microgametocytes were amoeboid in outline and 12.45 ± 2.58 *μ*M ×  3.74 ± 0.82 *μ*M in measurement and were loosely appressed with erythrocyte nucleus and envelope. The nucleus of the microgametocyte was submedian or median in position and measured 2.72 ± 0.63 *μ*M ×  1.71 ± 0.38 *μ*M. 16 to 19 small (17.85 ± 2.54 *μ*M) oval pigment granules were seen scattered randomly on the cytoplasm making it granular in appearance or sometimes were aggregated in dense clumps at the end of the parasite. The NDR was 0.7 ± 0.13. 3 to 42 parasites were seen per 100 microscopic fields. 

*Hosts*. *Aythya fuligula *(Tufted Duck), *Aythya nyroca* (Ferruginous Pochard), *Netta rufina* (Red-Crested Pochard), *Aythya ferina* (Common Pochard), *Anas strepera* (Gadwall), *Tadorna ferruginea* (Ruddy Shelduck), and *Anas acuta *(Northern Pintail).

*Locality*. Hakaluki haor (*n* = 9), Tanguar haor (*n* = 6).

*(5) Haemoproteus (Parahaemoproteus) nettionis*, Johnston and Cleland, 1909

*Morphology*. A total of seven birds belonging to three species were infected with both macro- and microgametocytes of *H. nettionis*. The cytoplasm of both gametes was homogenous in form, with occasionally some minute vacuoles. The gametocytes almost completely encircled the erythrocyte nucleus and markedly displaced laterally. Host cell was hypertrophied.

The macrogametocyte was dumbbellshaped with measurement of 15.75 ± 2.68 *μ*M ×  4.85 ± 0.64 *μ*M (Figure 2(b)). The macrogametocyte was closely appressed both to the nucleus and the envelope of erythrocytes. The nucleus of the parasite was compact and median in position with measurement of 4.5 ± 1.28 *μ*M × 3.33 ± 0.28 *μ*M. Pigment granules were roundish and ranged from 13 to 28 (20.25 ± 6.18). The NDR was 0.38 ± 0.05. Invasion intensity ranged from 2 to 35 parasites per 100 microscopic fields.

The microgametocytes were also dumbbell-shaped with 12.745 ± 4.8 *μ*M × 4.3 ± 0.26 *μ*M measurement. The parasite nucleus was submedian in position with 2.7 ± 0.58 *μ*M × 2.07 ± 0.5 *μ*M measurement. The microgametocytes have 17 to 19 (16 ± 1) small roundish pigment granules. The NDR was 0.4 ± 0.08. The invasion intensity was 4 to 16 parasites per 100 microscopic fields.

*Hosts*. *Aythya ferina* (Common Pochard), *Aythya nyroca *(Ferruginous Pochard), and *Anas acuta *(Northern Pintail).

*Locality*. Hakaluki haor (*n* = 5), Tanguar haor (*n* = 2).

*(6) Haemoproteus (Parahaemoproteus)* sp.

*Morphology*. Parasites of genus *Haemoproteus* of four birds of two species were identified up to subgenus level only. All of the parasites were macrogametocytes and were of oval shape. The gametes were laid closely with the host erythrocyte envelope. The dimension was 12.785 ± 1.47 *μ*M ×  3.595 ± 0.515 *μ*M. The nucleus was oval and measured 4.63 ± 0.9 *μ*M ×  2.56 ± 0.5 *μ*M. It was laid in the outer periphery of the macrogametocyte. The cytoplasm was coarsely granular with 25 to 29 roundish pigment granules. Host erythrocyte cell was slightly hypertrophied and the erythrocyte nucleus was slightly displaced laterally. The NDR was 0.9 ± 0.3. The invasion intensity was 3 to 9 per 100 microscopic fields.

*Hosts*. *Dendrocygna bicolor *(Fulvous Whistling Duck) and *Netta rufina* (Red Crested Pochard).

*Locality*. Hakaluki haor (*n* = 3), Tanguar haor (*n* = 1).

#### 3.2.2. Genus *Plasmodium, *Marchiafava and Celli, 1885

*(1) Plasmodium (Haemoamoeba) relictum*, Grassi and Feletti, 1891

*Morphology*. A total of 25 birds belonging to 17 species were infected with *P. relictum*. One was found to be infected with the microgamete and trophozoite of *P. relictum* and others were infected with only trophozoites. The microgamete was lobulated with oval pigment granules and markedly displaced the erythrocyte nucleus. The pigment granules were loosely clumped in the cytoplasm and the granules were 3.58 ± 0.05 *μ*M ×  2.06 ± 0.03 *μ*M in measurement. The invasion intensity was 5 parasites per 100 microscopic fields for the microgamete.

**Figure 3:**
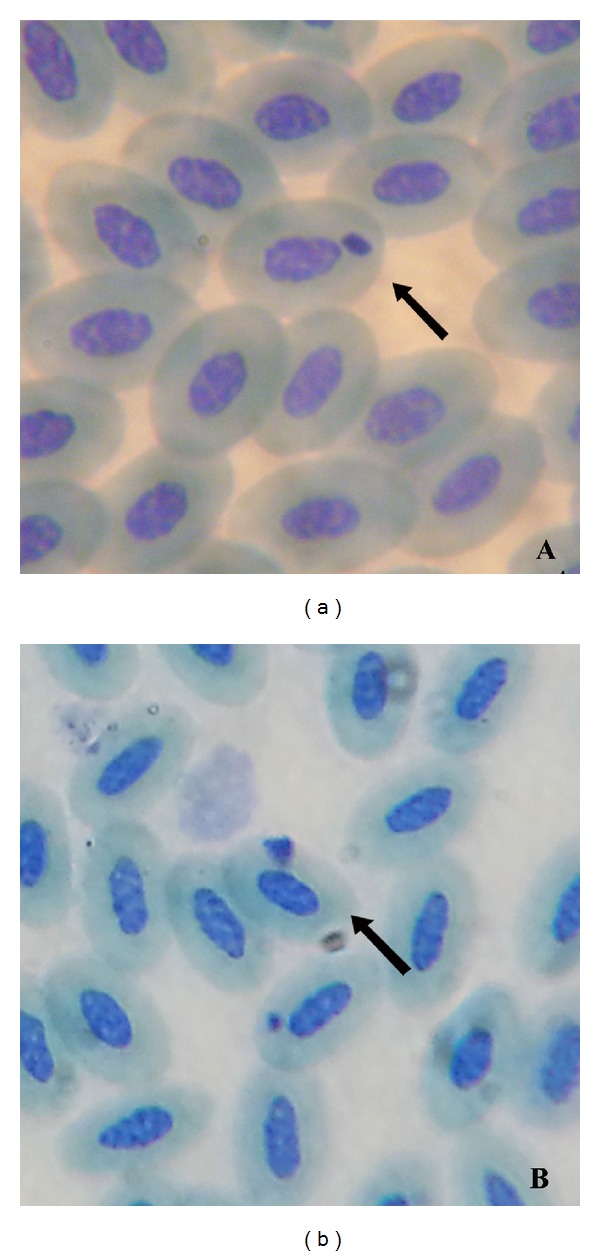
(a) Trophozoite of *P. relictum.* (b) Trophozoite of *P. lutzi*.

Some trophozoites were oval in shape and some were lobulated (Figure 3(a)). The trophozoites looked like solid bodies with relatively large nucleus. Roundish pigment granules were randomly dispersed in the cytoplasm. The measurements of the trophozoites were 3.54 ± 0.71 *μ*M ×  2.37 ± 0.7 *μ*M. Invasion intensity ranged from 2 to 19 per 100 microscopic fields.

*Hosts*. *Rostratula benghalensis *(Greater Painted Snipes), *Anas clypeata* (Northern Shoveler), *Anas penelope* (Eurasian Wigeon), *Aythya fuligula *(Tufted Duck), *Gallinura chloropus* (Common Moorhen), *Larus brunnicephalus* (Brown Headed Gull), *Anas querquedula *(Gargeney), *Tringa stagnatilis *(Marsh Sandpiper), *Fulica atra* (Common Coot), *Tringa totanus* (Common Redshank), *Dendrocygna bicolor* (Fulvous Whistling Duck), *Anas acuta *(Northern Pintail), *Todorna ferruginea* (Ruddy Shelduck), *Nycticorax nycticorax* (Black-Crowned Night Heron), *Vanellus cinereus* (Grey-headed Lapwing), *Netta rufina* (Red-Crested Pochard), and *Anas strepera* (Gadwall).

*Locality*. Hakaluki Haor (*n* = 13), Tanguar Haor (*n* = 12).

*(2) Plasmodium (Haemoamoeba) lutzi,* Lucena, 1939

*Morphology*. A total of nine birds belonging to seven species were found to be infected with *P. lutzi*. Trophozoites, situated in the subpolar or in polar position of the erythrocyte, were somewhat oval in shape with measurements of 3.67 ± 1.34 *μ*M × 2.5 ± 0.7 *μ*M (Figure 3(b)). The nucleus was comparatively large. Roundish to somewhat oval pigment granules were seen to be clumped near the margin of the trophozoites. When vacuoles were present they were few in number, measuring 1.03 ± 0.02 *μ*M. Infected erythrocyte was deformed and the erythrocyte nuclei were displaced markedly. Invasion intensity ranged from 2 to 12 parasites per 100 microscopic fields.

*Hosts*. *Fulica atra *(Common Coot), *Gallinura chloropus* (Common Moorhen), *Anas strepera* (Gadwall), *Ardeola grayii* (Indian Pond Heron), *Larus ridibundus* (Black-Headed Gull), *Porphyrio porphyrio* (Purple swamp hen), and *Anas acuta* (Northern Pintail).

*Locality*. Hakaluki haor (*n* = 4), Tanguar haor (*n* = 5).

*(3) Plasmodium (Giovannolaia) polare*, Manwell, 1934

*Morphology*. Three birds belonging to two species were found to be infected with *P. polarae*. In two birds somewhat oval shaped trophozoites were observed with a measurement of 3.3 ± 0.34 *μ*M × 3.35 ± 1.1 *μ*M. Several oval pigment granules were seen clumped in the cytoplasm. Vacuoles in the cytoplasm, when present, were plentiful. The trophozoites were in subpolar or polar position, occupying less than half of the erythrocyte. Invasion intensity was 4 for one bird and 7 parasites per 100 microscopic fields for the other bird.

Both types of gametes were seen in one bird and were lateral to the erythrocyte nucleus in position. Oval macrogamete with coarse oval pigment granules was seen. No vacuoles were seen. The macrogamete measured 3.575 ± 0.24 *μ*M ×  4.12 ± 0.1 *μ*M. Invasion intensity was 6 parasites per 100 microscopic fields. Microgamete was elongated in shape. It has oval pigment granules dispersed in the cytoplasm. The microgamete measured 7.21 ± 0.04 *μ*M ×  3.09 ± 0.23 *μ*M.

*Hosts*. *Mesophoyx intermedia *(Intermediate Egret) and *Dendrocygna bicolor* (Fulvous Whistling Duck).

*Locality*. Hakaluki haor (*n* = 3).

*(4) Plasmodium (Giovannolaia) circumflexum, *Kikuth, 1931

*Morphology*. A total of seven birds of five species were infected with *P. circumflexum*. Trophozoites were oval in shape measuring 3.5 ± 1.63 *μ*M × 2.84 ± 0.8 *μ*M. Pigment granules somewhat roundish to oval in shape were seen clumped at the edge of the trophozoites and sometimes dispersed in the cytoplasm. Several vacuoles were seen with an average diameter of 1.03 ± 0.01 *μ*M. Invasion intensity ranged from 5 to 18 parasites per 100 microscopic fields.

*Hosts*. *Nettapus coromandelianus* (Cotton Pygmy Goose), *Aythya fuligula* (Tufted Duck), *Aythya ferina *(Common Pochard), *Anas acuta* (Northern Pintail), and *Dendrocygna javanica* (Lesser Whistling Duck).

*Locality*. Hakaluki haor (*n* = 5), Tanguar haor (*n* = 2).

*(5) Plasmodium (Giovannolaia) lophurae*, Manwell, 1934

*Morphology*. A single bird was found to be infected with *P. lophurae*. Trophozoites were highly amoeboid in outline measuring 3.09 ± 0.02 *μ*M × 2.06 ± 0.04 *μ*M. Parasite nucleus was large with plentiful cytoplasm. Oval pigment granules are clumped in the cytoplasm. Several vacuoles were seen in trophozoites with average diameter of 1.03 ± 0.01 *μ*M. Intensity of invasion was 7 parasites per 100 microscopic field.

*Host*. *Larus ridibundus* (Black-Headed Gull).

*Locality*. Hakaluki haor (*n* = 1).

*(6) Plasmodium (Haemoamoeba)* sp., Grassi and Feletti, 1890

*Morphology*. Four birds of four different species were infected with trophozoites of *Plasmodium* genus. They were not identified up to species level due to the small number of parasites, but they were identified up to subgenus *Haemoamoeba*. In general, the trophozoites were elongated with oval pigment granules and dispersed in the cytoplasm, measuring 3.3 ± 0.8 *μ*M × 2.6 ± 0.2 *μ*M. They contained several vacuoles with a diameter of 1.03 ± 0.01 *μ*M.

*Hosts*. *Anas acuta *(Northern Pintail), *Aythya nyroca* (Ferruginous Pochard), *Anas querquedula* (Garganey), and *Anas strepera* (Gadwall).

*Locality*. Hakaluki haor (*n* = 2), Tanguar haor (*n* = 2).

*(7) Plasmodium (Giovannolaia)* sp., Corradetti, Garnham and Laird, 1963

*Morphology*. A single bird was infected with trophozoites of *Plasmodium* genus. This was identified up to Subgenus *Giovannolaia*. The merozoites were elongated with oval pigment granules, measuring 5.15 ± 0.02 *μ*M ×  2.575 ± 0.2 *μ*M. It did not contain vacuoles.

*Host*. *Podiceps cristatus *(Great Crested Grebe).

*Locality*. Tanguar haor (*n* = 1).

#### 3.2.3. Genus *Leucocytozoon *sp., Berestneff, 1904

**Figure 4:**
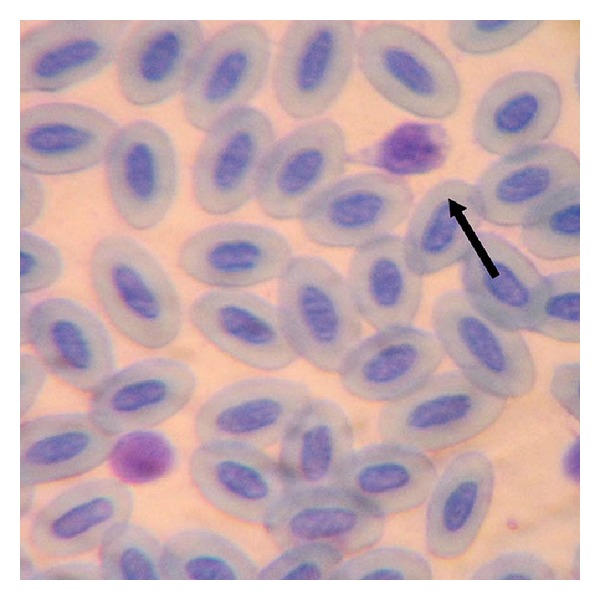
Macrogametocyte of *Leucocytozoon *sp.

*Morphology*. Only two birds of two species were positive for *Leucocytozoon *sp. (Figure 4). They were very few in number. All of them were macrogametocytes. They measured 10.8 ± 1.34 *μ*M × 8.7 ± 1.43 *μ*M. The dark blue cytoplasm with many small vacuoles appeared to be coarsely granulated. Small round organelles resembling the pigment granules were seen. The invasion intensity was 4 to 5 parasites per 100 microscopic fields.

*Hosts*. *Anas crecca *(Common Teal) and *Dendrocygna bicolor* (Fulvous Whistling Duck).

*Locality*. Hakaluki haor (*n* = 2).

#### 3.2.4. Microfilariae

**Figure 5:**
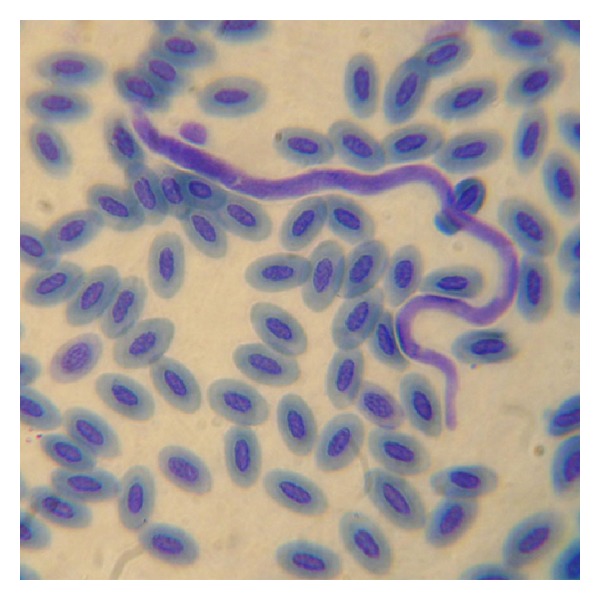
Microfilariae of genus *Paronchocerca. *

One unidentified species of microfilaria was reported from the blood of *Ardeola grayii *(Indian Pond Heron). It was caught from Hakaluki haor. The microfilaria is of *Paronchocerca *genus (Figure 5).

The worm was sheathed, long and cylindrical with length of 196 um and width of 3.5 *μ*m. The tail was bluntly rounded. Nuclei extend to posterior end of body, are loosely arranged. Intensity of invasion was 3 microfilariae per 100 microscopic fields.

## 4. Discussion

In this study, six species of *Haemoproteus, *seven species of *Plasmodium*, an unidentified species of *Leucocytozoon, *and an unidentified microfilarial species of genus *Paronchocerca *were reported in wild birds from different sites of Bangladesh. Although there are reports of *Leucocytozoon* sp. and *Haemoproteus* sp. in domestic pigeons [7] and ducks [6] in Bangladesh, but a comprehensive report on avian haematozoans is lacking. The findings of this study provide a comprehensive report.

According to the list of avian parasites prepared by Bennett [1] and Valkiunas [3], one bird can be invaded by several congeneric species of parasites and parasites of different taxa. In this study close to 45% of the examined bird species were infected with more than one congeneric species of parasite or parasites of different taxa.

A lower prevalence of *Haemoproteus *(13.1%), *Plasmodium *(15.1%) and *Leucocytozoon* (0.6%) genera was observed here than in studies in India and Myanmar, which are neighboring countries of Bangladesh. In India 18% prevalence was reported for *Haemoproteus *and 28% for *Plasmodium* in wild birds and in Myanmar 40% for *Haemoproteus *and 60% for *Plasmodium *in wild birds [11]. Their reported prevalence was higher, likely because they used molecular methods to detect parasites, which are known to be more sensitive. Another reason for the low prevalence found in this study may be that we sampled during winter (January to March), which is not the breeding season of lowland resident birds [11] and there are low levels of sexual steroid hormones circulating in the birds' blood, which are supposed to allow the parasite to survive [21, 22].

The intensity of invasion for parasites varied remarkably. Most of the invasions for *Haemoproteus *were below 20 parasites per 100 microscopic fields and invasions for *Plasmodium *were below 10. This verifies the proposition of Valkiunas [23] that it is difficult to capture heavily invaded birds using the mist net method as they are less active than the less heavily invaded birds [23]. The intensity of invasion per 100 microscopic fields was higher in *Haemoproteus* than in *Plasmodium *and *Leucocytozoon*. *Haemoproteus *gametocytes persist in the peripheral blood for a long time [15], while some species of *Leucocytozoon *prefer visceral circulation [24]; therefore, *Leucocytozoon *may have escaped our attention. In contrast to *Haemoproteus *and *Leucocytozoon*, for parasites of *Plasmodium *genus, though they prefer peripheral blood circulation, the schizogonic cycle in the erythrocytes lasts for only few days [3]. As a result they may also have escaped our attention.

Variations in the prevalence of infection in different bird families have been reported in this study, a finding supported by different studies around the globe [11, 25–28].

The report of microfilarial infection in birds in Bangladesh is perhaps the first. The prevalence of microfilariae in Bangladeshi birds appears to be low. The intensity of infection was also low, which may be an effect of the absence of microfilariae in the peripheral blood stream in the daytime due to their periodicity (sampling was done in the daytime) [29, 30]. It is assumed to be nonpathogenic to birds [31–33]. It may have some histopathological impact but no cellular reaction [34]. However, microfilarial infection may lead to questionable fitness of the host bird [35].

In our study, more migratory birds were infected than resident birds. This finding is supported by some other studies [26, 27]. In different studies there are indistinct data on this issue due to different host species composition [36]. As migratory birds travel in the course of different terrain they increase their affinity for diverse parasite vectors, which may lead to increased chance of infection in migratory birds [3, 37, 38]. Migrants can play a vital role in transmitting parasites among taxonomically similar birds from temperate regions to tropical regions, which has been observed in Nigeria [39].

## 5. Conclusion

The pathogenic effects of these parasites can play a vital role in declining wildlife population [40]. Wild birds of Bangladesh carry several types of hemoprotozoan parasites. Further investigation with a larger sample size is necessary to estimate more accurately the prevalence of haematozoan parasites among wild birds and to understand the epidemiology of those parasites.

## Supplementary Material

Accession numbers of the Parasite slides deposited in U.S. National Parasite Collection (USNPC), Beltsville, MD 20705, USA

## Figures and Tables

**Table 1:** Number of the examined birds, their migration status and blood parasite prevalence (in %) in different bird families and species.

Birds families/ species	Migration status	Number of examined birds	Genus *Haemoproteus *		Genus *Plasmodium *		Genus *Leucocytozoon *	
			Prevalence (%)	Parasite species	Prevalence (%)	Parasite species	Prevalence (%)	Parasite species
Anatidae		**140**	**17.9**		**16.4**		**0.7**	
*Anas acuta *	M	32	9.4	*H. greineri *(*2*), *H. nettionis *	18.8	*P. *(*G*)* circumflexum *(*2*), *P. *(*H*)* lutzi,* *P. *(*H*)* relictum *(*2*), *P. *(*Haemamoeba*) sp.		
*Anas clypeata *	M	3			66.7	*P. *(*H*)* relictum *(*2*)		
*Anas crecca *	M	4					25.0	*Leucocytozoon* sp.
*Anas falcata *	M	1						
*Anas penelope *	M	2			50.0	*P. *(*H*)* relictum *		
*Anas querquedula *	M	7			28.6	*P. *(*H*)* relictum,* *P. *(*Haemamoeba*) sp.		
*Anas strepera *	M	12	8.3	*H. greineri *	25.0	*P. *(*H*)* lutzi,* *P. *(*H*)* relictum,* *P. *(*Haemamoeba*) sp.		
*Aythya ferina *	M	10	50.0	*H. greineri *(*2*), *Mixed H. greineri *and *H. nettionis *(*1*), *H. nettionis *(*2*)	10.0	*P. *(*G*)* circumflexum *		
*Aythya fuligula *	M	26	3.8	*H. greineri *	15.4	*P. *(*G*)* circumflexum *(*2*), *P. *(*H*)* relictum *(*2*)		
*Aythya nyroca *	M	14	42.9	*H. greineri *(*3*)*, H. nettionis *(*3*)	7.1	*P. *(*Haemamoeba*) sp.		
*Netta rufina *	M	4	75.0	*H. greineri *(*2*), *H. *(*Parahaemoproteus*) sp*. *	25.0	*P. *(*H*)* relictum *		
*Nettapus coromandelianus *	R	9			11.1	*P. *(*G*)* circumflexum *		
*Tadorna ferruginea *	M	13	23.1	*H. greineri *(*3*)	7.7	*P. *(*H*)* relictum *		
*Tadorna tadorna *	M	3	100.0					

Dendrocygnidae		**43**	**7.0**		**9.3**		**2.3**	
*Dendrocygna bicolor *	R	36	8.3	*H. *(*Parahaemoproteus*) sp. (*3*)	8.3	*P. *(*G*)* polarae, * *P. *(*H*)* relictum *(*2*)	2.8	*Leucocytozoon* sp.
*Dendrocygna javanica *	R	7			14.3	*P. *(*G*)* circumflexum *		

Accipitridae		**1**						
*Circus melanoleucos *	M	1						

Ardeidae		**15**			**20.0**			
*Ardea cinerea *	R	1						
*Ardeola grayii *	R	8						
*Bubulcus ibis *	R	1						
*Egretta garzetta *	R	1						
*Mesophoyx intermedia *	R	2			100.0	*P. *(*G*)* polarae *		
*Nycticorax nycticorax *	R	2			50.0	*P. *(*H*)* relictum *		

Charadriidae		**8**	**12.5**		**12.5**			
*Charadrius dubius *	R	6	16.7	*H. scolopaci *				
*Vanellus cinereus *	M	2			50.0	*P. *(*H*)* relictum *		

Jacanidae		**4**						
*Hydrophasians chirurgus *	R	4						

Laridae		**34**	**44.1**		**8.8**			
*Chlidonias hybridus *	M	1						
*Larus brunnicephalus *	M	7			28.6	*P. *(*H*)* relictum *(*2*)		
*Larus ridibundus *	M	26	57.7	*H. plataleae *(*15*)	3.9	*P. *(*G*)* lophurae, *		

Phalacrocoracidae		**1**						
*Phalacrocorax carbo *	M	1						

Podicipedidae		**4**			**25.0**			
*Podiceps cristatus *	M	2			50.0	*P. *(*Giovannolaia*) sp.		
*Tachybaptus ruficollis *	R	2						

Rostratulidae		**2**			**50.0**			
*Rostratula benghalensis *	M	2			50.0	*P. *(*H*)* relictum *		

Scolopacidae		**24**			**20.8**			
*Calidris alpina *	M	2						
*Calidris minuta *	M	10						
*Calidris temminckii *	M	1						
*Gallinago stenura *	M	1						
*Limnodromous semipalmatus *	M	1						
*Tringa stagnatilis *	M	2			50.0	*P. *(*H*)* relictum *		
*Tringa totanus *	M	7			57.1	*P. *(*H*)* relictum *(*4*)		

Rallidae		**38**			**18.4**			
*Fulica atra *	M	32			15.6	*P. *(*H*)* lutzi *(*3*), *P. *(*H*)* relictum *(*2*)		
*Gallicrex cinerea *	R	2						
*Gallinura chloropus *	M	1			100.0	*P. *(*H*)* lutzi *		
*Porphyrio porphyrio *	R	3			33.3	*P. *(*H*)* lutzi *		

Sturnidae		**2**	**50.0**					
*Sturnus contra *	R	2	50.0	*H. pastoris *				

Sylviidae		**2**						
*Acrocephalus stentoreus *	M	1						
*Megalurus palustris *	R	1						

Eurostopodidae		**1**						
*Eurostopodus macrotis *	R	1						

Total		319	13.2		15.1		0.6	

Migration status: M: migrant, R: resident.
