# Supplementary material for: Prevalence and Diversity of Avian Haematozoan Parasites in Wetlands of Bangladesh
Source: J Parasitol Res. 2014 Jan 21;2014:493754. doi: 10.1155/2014/493754 (PMC3918735; doi:10.1155/2014/493754)
Supplement: Supplementary file 1 — Accession numbers of the Parasite slides deposited in U.S. National Parasite Collection (USNPC), Beltsville, MD 20705, USA [file 493754.f1.pdf]

Supplementary file 1: Accession numbers of the Parasite slides deposited in U.S. National Parasite Collection (USNPC), Beltsville, MD 20705, USA

USNPC 107319.00	*Haemoproteus pastoris*
USNPC 107320.00	*Haemoproteus scolopaci*
USNPC 107321.00	*Haemoproteus plataleae*
USNPC 107322.00	*Haemoproteus greineri*
USNPC 107323.00	*Haemoproteus nettionis*
USNPC 107324.00	*Plasmodium (Haemoamoeba) relictum*
USNPC 107325.00	*Plasmodium (Haemoamoeba) lutzi*
USNPC 107326.00	*Plasmodium (Giovannolaia) polare*
USNPC 107327.00	*Plasmodium (Giovannolaia) circumflexum*
USNPC 107328.00	*Plasmodium (Giovannolaia) lophurae*
USNPC 107329.00	*Leucocytozoon* sp.
USNPC 107330.00	*Paronchocerca* sp.
